# From Social Media Content to Value Co-Creation: Role of Environmental Attitude, Environmental Knowledge, and Green Truth

**DOI:** 10.3390/foods15071120

**Published:** 2026-03-24

**Authors:** Gabriel Usiña-Báscones, Nelson Carrión-Bósquez, Mayra Samaniego-Arias, Rubén Marchena-Chanduvi, Santiago Medina-Miranda, Wilson Zambrano-Vélez, Wilfredo Ruiz-García, Mary Llamo-Burga, Oscar Ortiz-Regalado

**Affiliations:** 1 Facultad de Ciencias Sociales, Educación Comercial y Derecho, Facultad de Posgrados/Programa de Posgrados, Universidad Estatal de Milagro, Milagro 091050, Ecuador; msamaniegoa@unemi.edu.ec; 2 Departamento de Administración, Facultad de Economía y Administración, Universidad Católica del Norte, Antofagasta 1270709, Chile; 3 Escuela Profesional de Ingeniería Agroindustrial, Facultad de Ciencias Agrarias, Universidad Nacional Autónoma de Chota, Cajamarca 06001, Peru; rmarchenac@unach.edu.pe; 4 Escuela Profesional de Ingeniería en Agronegocios, Escuela Profesional de Agronomía, Universidad Nacional de Cajamarca, Cajamarca 06001, Peru; smedina@unc.edu.pe (S.M.-M.); wruizg@unc.edu.pe (W.R.-G.); oortizr@unc.edu.pe (O.O.-R.); 5 Facultad de Ciencias Sociales y de la Salud, Universidad Estatal Península de Santa Elena, La Libertad 240250, Ecuador; wzambrano@upse.edu.ec

**Keywords:** social media content, value co-creation, organic food consumption, environmental attitude, environmental awareness, green trust, sustainable consumption, PLS-SEM

## Abstract

This study examined how social media content influences value co-creation among organic product consumers through the mediating roles of environmental awareness, green truth, and environmental attitude. Grounded in the Stimulus-Organism-Response (SOR) framework, social media content is conceptualized as a stimulus, environmental awareness, green trust, and environmental attitude as internal organism states, and value co-creation as the behavioral response. A cross-sectional quantitative design was applied using a 20-item questionnaire administered to 739 organic-product consumers. Data were analyzed using partial least-squares structural equation modeling (PLS-SEM). The results indicate that social media content does not directly affect value co-creation but significantly influences environmental awareness, green trust, and environmental attitude. Environmental awareness and green trust positively affect both environmental attitude and value co-creation, and environmental attitude emerges as the strongest direct predictor of value co-creation. These findings confirm the mediating role of cognitive and attitudinal mechanisms in transforming digital sustainability content into collaborative consumer behavior. This study contributes to the literature on sustainable consumption by integrating communication, cognitive, and attitudinal variables in a single explanatory model. Practically, the findings suggest that sustainability communication strategies in digital environments should prioritize credibility and environmental knowledge to foster consumer participation in value co-creation.

## 1. Introduction

Social media has become one of the main digital environments in which consumers access information, interact with brands and other users, and construct meanings around products, lifestyles, and values associated with sustainability [1,2,3]. In the context of organic product consumption, these platforms play a key role in shaping perceptions, beliefs, and environmental attitudes (EAT) through continuous exposure to informational content, experiences, and norms related to health and environmental care [4,5,6]. In this regard, the literature shows that Social Media Content (SMC) can shape organic consumer behavior by reinforcing pro-environmental values, healthy lifestyles, and ethical consumption criteria, especially when such content is perceived as relevant and credible [7,8].

The growing interest in organic products stems from the need to mitigate the negative impacts of the conventional agri-food system on the environment and human health, positioning these products as sustainable alternatives aimed at reducing chemical use, conserving ecosystems, and promoting social well-being [9]. Therefore, the consumption of organic products is not solely explained by functional benefits but also by ethical and environmental motivations that reflect a higher level of awareness regarding individual and collective responsibility in preserving the planet [10,11]. This is particularly evident in organic consumption markets, especially in emerging economies, where organic product consumers tend to demonstrate greater environmental sensitivity and a stronger willingness to become actively involved in practices that promote sustainable development, thus surpassing the traditional act of purchase [12].

Within this context, Value Co-creation (VCC) emerges as a central approach to understanding how consumers actively participate in generating value together with organizations through processes of interaction, knowledge exchange, and continuous collaboration in the dissemination of sustainability-related messages [13,14]. In sustainable consumption contexts, VCC enables consumers to contribute ideas, feedback, and promote responsible practices, thereby strengthening initiatives aimed at environmental and social well-being [15,16]. Accordingly, the literature indicates that VCC is particularly relevant in markets, such as organic products, where shared values and EAT encourage collaborative behaviors and the development of conscious consumption communities [17,18].

In light of the above, it is necessary to conceptualize the variables that structure the dynamics of VCC. First, SMC is defined as the set of messages, images, and videos generated by firms and users that influences consumers’ perceptions and evaluations [1,3]. Moreover, Environmental Awareness (EAW) refers to the level of understanding and sensitivity individuals have toward ecological problems and their consequences, constituting a key antecedent of pro-environmental attitudes [19,20]. Likewise, Green Truth (GTR) is conceptualized as the perceived credibility, authenticity, and consistency of communicated environmental information, influencing consumer trust and the effectiveness of green messages disseminated in digital environments [5,21,22,23]. Finally, EAT refers to an individual’s cognitive, affective, and behavioral predisposition toward environmental protection, representing a determining factor in sustainable behavior and participation in environmental protection processes [24,25].

Despite the empirical evidence provided by some studies on sustainable consumption and digital communication, a gap remains in the integrated understanding of how SMC influences VCC through attitudinal and cognitive mechanisms among organic product consumers [26,27]. In particular, there is limited empirical evidence explaining how variables such as EAW, GTR, and EAT act as antecedents that facilitate the transformation of digital stimuli into collaborative behaviors oriented toward sustainability [12,23]. In this context, this study is justified by the need to integrate these variables into an explanatory model that enables a better understanding of the factors that promote VCC among organic product consumers, thereby providing relevant empirical evidence for the design of communication strategies, environmental education initiatives, and sustainable consumption management.

This study focuses on young consumers, primarily university students, who represent an increasingly relevant segment of the organic product market [7,8,28]. Despite the growing body of research examining the influence of social media on consumer behavior and sustainable consumption, important gaps remain in understanding the mechanisms through which social media communication shapes consumer participation in value co-creation processes. While previous studies have explored social media engagement and green consumption separately, limited research has integrated these perspectives within a unified theoretical framework that explains how SMC stimulate psychological evaluations and collaborative consumer behaviors. To address this gap, the present study adopts the Stimulus-Organism-Response (SOR) model to examine how SMC influences consumers’ EAT, EAW, and GTR, ultimately shaping their willingness to engage in VCC within the context of organic product consumption. To provide empirical evidence and address existing knowledge gaps, this study aims to identify the role of EAT, EAW, and GTR in the relationship between SMC and VCC among organic product consumers. To further support this objective, the following research questions are proposed: (a) How does SMC influence the VCC? (b) How does SMC influence the EAT, EAW, and GTR? (c) How does the EAW influence the VCC and EAT? (d) How does GTR influence VCC and EAT? (e) How does EAT influence the VCC?

## 2. Literature Review

### 2.1. Stimulus-Organism-Response Model

The processes through which environmental stimuli influence consumers’ attitudes and behaviors have traditionally been addressed from perspectives that emphasize the economic, social, and institutional structures of value creation [27]. However, these frameworks tend to present limitations in explaining how certain external factors interact with individuals’ internal states to generate specific behavioral responses, particularly in dynamic contexts such as digital environments and sustainable consumption [8]. In response to these limitations, the Stimulus-Organism-Response (SOR) model broadens the analysis by integrating factors that function as external stimuli shaping individuals’ cognitive and affective processes, thereby offering a more comprehensive perspective capable of examining the formation of attitudes and behaviors in scenarios of complex social and communicative interactions [28,29].

Within the SOR framework, stimuli represent environmental cues that trigger internal cognitive and affective processes. In the context of social media, these stimuli take the form of communication signals integrated into digital content, including informative posts, promotional messages, sustainability narratives, and user-generated content related to organic products [28]. Rather than focusing on specific platform features or message formats, this study conceptualizes SMC as the perceived exposure to sustainability-related information circulating within social media ecosystems [8]. This perception-based operationalization is consistent with previous applications of the SOR model in digital consumer research, where the psychological impact of perceived content exposure is considered to be the relevant stimulus influencing consumers’ internal evaluations and subsequent behavioral responses.

From this perspective, the SOR model is particularly suitable for analyzing the impact of SMC on consumer behavior as it conceptualizes digital messages as stimuli capable of activating internal processes that mediate behavioral responses [8,30]. In the context of organic product consumption, content disseminated through social media functions as an external stimulus that influences cognitive and attitudinal variables such as EAW, perceived GTR, and EAT, which together shape the consumer’s organism and determine their willingness to engage in collaborative behaviors oriented toward sustainability [31,32]. Consequently, the response is manifested through VCC, understood as the consumer’s active participation in the processes of exchange, collaboration, and shared value generation, thereby reinforcing the relevance of the SOR model as an integrated framework to explain the stimulus-processing-response sequence in digital environments of sustainable consumption [18,33,34].

### 2.2. Social Media Content

SMC is understood as a set of materials created and shared on social platforms (text, images, videos, audiovisual productions, multimedia, links, or other forms of content) that can be viewed and interacted with by other users, thereby influencing the processing and evaluation of messages in digital environments [1,2,3]. In this sense, the SMC construct captures respondents’ perceived exposure to sustainability-related SMC, rather than objective measures of platform use or content format [8,28]. In this sense, such content becomes relevant within consumer behavior contexts because, when it is informative, engaging, and perceived as authentic, it can shape perceptions and beliefs about the environmental impact of consumption, reinforcing pro-environmental orientations linked to the organic product market [4,5,7]. Consequently, analyzing SMC as a stimulus is essential for understanding how consumers interpret sustainability cues and how these cues can activate internal dispositions that are later expressed in behavioral responses associated with responsible consumption [8,29].

From a theoretical perspective, SMC has been associated with participation and interaction dynamics that facilitate the exchange of resources, knowledge, and experiences, which is consistent with the logic of message diffusion in digital ecosystems [4,14,33]. The literature argues that digital environments can stimulate participatory responses aimed at generating shared value, as symbolic and affective interactions on social platforms encourage collaboration and engagement with sustainability initiatives [18,30,34]. Consequently, it is important to investigate whether and under what conditions SMC acts as an antecedent that directly drives VCC among organic product consumers, given that integrative frameworks are still needed to explain how digital stimuli are transformed into observable collaborative behaviors [18,27]. In light of this, this study sought to test the following hypothesis:

**H1a.**  *Social Media Content influences Value Co-creation.*

On the other hand, academic literature has established that frequent exposure to environmental content on social media is associated with a greater appreciation of sustainability and with the strengthening of predispositions toward environmental protection, which is directly connected to the formation of favorable attitudes toward protecting the environment [6]. However, this influence depends on conditions such as source credibility and the perceived environmental effectiveness of the message, as users respond more positively when they consider the information trustworthy and the cause relevant [22]. Therefore, the literature also acknowledges gaps regarding which types of content (emotional versus informational and user-generated versus firm-generated) are more effective in transforming attitudes and behaviors, which justifies further empirical examination of the relationship between SMC and EAT in the context of organic product consumption [5,35]. In light of this, this study sought to test the following hypothesis:

**H1b.**  *Social Media Content influences Environmental Attitude.*

The EAW is strengthened when individuals access information that increases their understanding of ecological problems, consequences, and possible solutions. In this process, social media can operate as channels to expand access to educational content and promote flexible learning at the scale [36,37]. Moreover, the literature has shown that exposure to environmental information on social media can increase the intention to engage in pro-environmental behaviors by reinforcing internal perceptions and orientations related to environmental care, suggesting a mechanism of influence that should be modeled together with other cognitive factors [38]. Consequently, given that questions remain about how content type, source credibility, and emotional framing interact with prior knowledge and cultural context, it is necessary to investigate in an integrated manner the link between SMC and EAW among organic product consumers in [36,39]. In light of this, this study sought to test the following hypothesis:

**H1c.**  *Social Media Content influences Environmental Awareness.*

In digital environments, the effectiveness of green content depends on the trust users place in the platform and source, as well as on their evaluation of the message’s perceived environmental effectiveness, factors that shape how beliefs are formed, and how sustainability-related images are consolidated [5]. Likewise, studies indicate that user-generated content can have a particular impact on the formation of perceptions owing to its association with authenticity and proximity, which is relevant for understanding how judgments of truthfulness and coherence regarding environmental messages are constructed [22]. In this regard, considering that green communication and engagement on social media can foster the transition toward the adoption of sustainable actions, it is justified to investigate the influence of SMC as a necessary condition that may strengthen or weaken beliefs in messages that share information on sustainability issues [23]. In light of this, this study sought to test the following hypothesis:

**H1d.**  *Social Media Content influences Green Truth.*

### 2.3. Environmental Awareness

EAW can be understood as a state of sensitivity and understanding of ecological problems and the consequences of human actions, which is strengthened through processes of environmental literacy grounded in knowledge, values, and pro-environmental behaviors [40]. In this sense, EAW is consolidated when individuals not only incorporate facts and concepts about natural systems but also recognize the practical relevance and effectiveness of responsible behaviors in everyday life [41,42]. Consequently, within the context of organic product consumption, EAW is critical because it serves as a cognitive and value-based foundation for interpreting sustainability messages, evaluating consumption alternatives, and guiding predispositions toward practices aligned with environmental protection [37,38]. Therefore, its inclusion in the study model is relevant for understanding how digital stimuli can activate internal mechanisms that subsequently foster collaborative responses associated with sustainable consumption [8,29].

Empirical evidence on EAW indicates that higher levels of environmental literacy and awareness promote participation in collective initiatives and collaborative dynamics oriented toward sustainability, which is consistent with the logic of generating shared value among consumers, organizations, and communities [18,34]. Thus, the exchange of resources, knowledge, and environment-related interactions is strengthened when consumers possess a more conscious environmental orientation that motivates them to actively engage in shared and sustainable solutions [13,14]. Likewise, studies on sustainable consumption suggest that pro-environmental orientations increase the willingness to participate in responsible initiatives and practices that go beyond purchasing, reinforcing the need to empirically examine whether EAW functions as an antecedent of VCC among organic consumers [15,18]. Thus, investigating this relationship is relevant for understanding how environmentally aware consumers can become active agents contributing to sustainable value generation in market ecosystems [16,34]. In light of this, this study sought to test the following hypothesis:

**H2a.**  *Environmental Awareness influences Value Co-creation.*

Regarding the antecedents linking EAW and EAT, the literature shows that increases in environmental understanding and learning tend to translate into more favorable predispositions toward environmental protection, strengthening pro-environmental beliefs, emotions, and behavioral intentions [20,43]. In this way, EAT is recognized as integrating cognitive and affective dimensions, so awareness and knowledge-based sensitization processes operate as antecedents for consolidating favorable evaluations of sustainability [24,25]. Moreover, in digital environments, exposure to environmental information on social media can increase the intention to engage in pro-environmental behaviors by reinforcing internal states related to awareness and perceived control, suggesting a pathway linking EAW with the formation of EAT [38,39]. Consequently, given that gaps remain regarding the specific mechanisms through which EAW is transformed into EAT in organic consumption contexts within emerging economies, it is necessary to further empirically examine this relationship within the proposed model [26,39]. In light of this, this study sought to test the following hypothesis:

**H2b.**  *Environmental Awareness influences Environmental Attitude.*

### 2.4. Green Truth

GTR can be conceptualized as the perceived credibility, authenticity, and coherence of environmental messages circulating in digital environments, especially when consumers evaluate whether green content is trustworthy and reflects genuine environmental commitment [22]. In this sense, GTR is closely related to trust in the source and to the evaluation of the message’s perceived environmental effectiveness, since users tend to respond more positively when they consider the content credible and the environmental cause relevant [5]. Consequently, its importance in the context of this study lies in the fact that in markets such as organic products (where consumers seek sustainability cues), the perceived truthfulness of content can shape how information is interpreted, how pro-environmental values are embraced, and how willing consumers engage in practices aligned with responsible consumption [8,10]. Therefore, it is important to analyze how GTR can activate internal states that support behavioral responses oriented toward sustainability [29].

The literature suggests that trust and credibility in communication can foster consumer participation and engagement in collaborative dynamics, as message diffusion can rely on interactions, knowledge exchange, and voluntary contributions within digital ecosystems [14,33]. In turn, when green messages are perceived as authentic, consumers tend to strengthen the formation of positive perceptions, which may increase their willingness to share experiences, recommend sustainable practices, and collaborate with brand- or community-related initiatives [22,34]. Moreover, since VCC is a multidimensional process that integrates direct and indirect interactions, as well as shared resources [13], GTR may constitute a relevant antecedent explaining why consumers decide to engage in collaborative actions aimed at generating sustainable value [44]. Therefore, it is necessary to empirically investigate the relationship between GTR and VCC among organic product consumers, particularly given that integrative frameworks are still required to clarify the psychological and contextual mechanisms that transform green communication into observable collaborative behaviors [18,27]. In light of the above, the study tested the following hypothesis:

**H3a.**  *Green Truth Influences Value Co-creation.*

On the other hand, there is evidence that social media can influence the formation of pro-environmental attitudes, although its effectiveness depends on factors such as source credibility and trust in the platform, which directly connect to the logic of GTR [5,6]. Complementarily, exposure to environmental content on social media has been associated with greater appreciation of sustainability, especially when the message is perceived as relevant and trustworthy, thereby reinforcing favorable predispositions toward environmental protection [22,35]. Furthermore, since EAT comprises cognitive and affective components, the perception of authenticity and coherence in green messages can strengthen beliefs and emotions related to environmental care, contributing to the consolidation of pro-environmental behavioral dispositions [24,25]. Considering that the literature recognizes gaps regarding the effectiveness of specific messages or information sources in transforming attitudes, this study justifies the need to analyze GTR as a key factor in understanding how environmental communication on social media translates into stronger ETR within organic consumption contexts [6,35]. In light of the above, the study tested the following hypothesis:

**H3b.**  *Green Truth influences environmental attitudes.*

### 2.5. Environmental Attitude

EAT is conceptualized as an individual’s predisposition to evaluate, feel, and act favorably or unfavorably toward the environment, integrating beliefs, emotions, and behavioral intentions related to its protection [24]. In this sense, EAT comprises a cognitive dimension associated with knowledge and beliefs about ecological problems, an affective dimension linked to emotions toward nature, and a behavioral dimension related to willingness to act in favor of the environment [25]. Consequently, the relevance of EAT in the present study lies in its role as a key internal state that translates external stimuli into sustainable orientations that influence organic product consumption and participation in responsible actions [6,8]. Moreover, evidence indicates that exposure to environmental messages on social media is associated with greater appreciation of sustainability, although its effect depends on conditions such as source credibility, reinforcing the need to understand how EAT is consolidated in digital environments [5,35]. Therefore, investigating EAT as a central variable in the model helps explain the mechanism by which cognitive and communicative factors are transformed into observable responses within organic consumption contexts [29,43].

The literature acknowledges that individuals with stronger pro-environmental orientations show a greater willingness to engage in collaborative initiatives aimed at sustainability and actively participate in processes of exchange, recommendation, and shared value generation [15]. In this regard, empirical evidence suggests that pro-environmental attitudes are associated with supportive behaviors toward sustainable initiatives and the co-production of solutions oriented toward environmental care, reinforcing the relevance of modeling EAT as a direct antecedent of VCC [18,34]. Therefore, given that more robust explanations are still needed for how EAT translates into concrete co-creation behaviors across different contexts and stakeholder groups, it is necessary to further examine this relationship empirically among organic product consumers [18,27]. In light of the above, the present study sought to test the following hypothesis:

**H4.**  *Environmental attitudes influence Value Co-creation among consumers of organic products.*

### 2.6. Hypothesized Model

**Figure 1:**
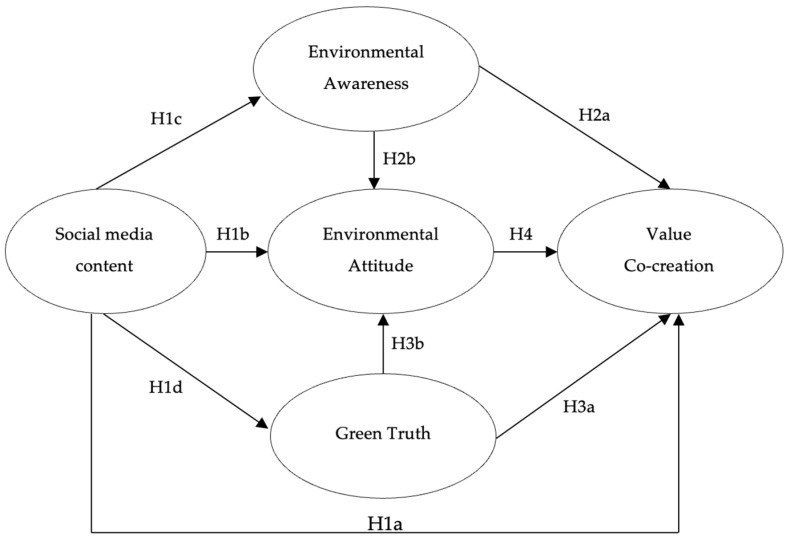
Research model.

The research model and its corresponding hypotheses to be tested are presented below (See Figure 1).

## 3. Methodology

### 3.1. Instrument Design and Data Collection

To empirically assess the validity of the proposed model, a cross-sectional quantitative correlational study was conducted using a 20-item questionnaire adapted to Spanish. The instrument was administered to consumers who were identified as buyers of organic products. Prior to data collection, the survey was reviewed by two experts in Marketing and Research Methodology who confirmed the adequacy of the items and raised no objections. After expert validation, a pilot test was conducted with 30 participants to ensure clarity and comprehensibility of the questions. The final sample consisted of 739 valid responses from 761 distributed questionnaires, yielding an effectiveness rate of 97%. Data were collected through a self-administered online survey, and all responses were carefully screened to exclude incomplete and inconsistent answers. This study employed convenience sampling, targeting university students who had previously consumed organic products. Undergraduate and graduate students are considered an appropriate group for examining social media-driven consumption behaviors, as younger consumers tend to be more exposed to digital communication environments and sustainability-related content [8,9,10,11,12,13,14,15,16,17,18,19,20,21,22,23,24,25,26,27,28]. Therefore, the sample represents a segment of young organic consumers rather than an entire population of organic food buyers [7,12,26]. Inclusion criteria required participants to be at least 18 years old and have purchased organic products during the previous three months. The data were collected using Google Forms. The structural model assessment and validation were conducted using SmartPLS 4.1.1.7.

### 3.2. Measures

The items for each construct were adapted from the previously validated measurement scales. A seven-point Likert scale was used in this study. The questionnaire included four items to measure each variable included in the model. Items measuring SMC and VCC were adapted from Li et al. [45], items for EAW and EAT were adapted from Trivedi et al. [46], and the items used to assess GTR were adapted from Chen and Chang [47]. It is important to note that the initial measurement instrument consisted of 20 items (four indicators per construct), but during the measurement model assessment, two indicators (EAT4 and VCC4) were removed because of low outer loadings and their negative impact on construct reliability and convergent validity. The final model included 18 indicators.

### 3.3. Statistical Procedure

Partial least squares structural equation modeling (PLS-SEM) was employed to estimate and evaluate the proposed model. The hypothesized model was estimated using this statistical technique, and its evaluation followed the methodological guidelines and recommended threshold values established by Hair et al. [48]. Unlike covariance-based SEM (CB-SEM), PLS-SEM does not require strict assumptions regarding residual distributions, allows specification of both formative and reflective measurement models, and is particularly suitable for moderate sample sizes. Furthermore, it is appropriate to analyze complex models that integrate multiple theoretical constructs with empirical data. In line with the methodological recommendations of Leguina [49], a two-step analytical approach was adopted. First, the measurement model was assessed to verify internal consistency and convergent validity by analyzing item factor loadings, Cronbach’s alpha values, Composite Reliability, and Average Variance Extracted. Discriminant validity was assessed using the Fornell–Larcker criterion and heterotrait–monotrait (HTMT) ratio. In the second step, the structural model was evaluated and the proposed hypotheses were tested using PLS-SEM, examining the statistical significance of the hypothesized relationships, path coefficients (β), Standardized Root Mean Square Residual (SRMR), and coefficients of determination (R^2^).

## 4. Results

### 4.1. Demographic Results

The study sample consisted of 739 consumers who reported having purchased organic products during the previous three months. The demographic profile of the respondents showed a higher proportion of women (58%), and the predominant age range was between 23 and 34 years (68%). In addition, 67% of participants reported having completed their undergraduate studies. The geographic distribution of respondents was highest in Santo Domingo de los Colorados (28%), followed by Quito (25%) and Guayaquil (21%). Table 1 presents a detailed description of the study’s demographic findings, and Appendix A.1 shows the cities in which the study was conducted.

### 4.2. Measurement Model Assessment (Convergent and Discriminant Validity of the Model)

Following the guidelines for PLS-SEM measurement evaluation (Hair et al., 2022 [50]), indicators with insufficient outer loadings were examined and removed when they weakened the construct reliability and convergent validity. Specifically, items EAT4 and VCC4 exhibited relatively low loadings, which reduced the composite reliability of their respective constructs. Therefore, these indicators were excluded in order to improve the measurement quality of the model. Although two indicators were removed, the remaining items retained adequate conceptual coverage of the constructs, as each construct continued to be represented by multiple indicators capturing its core theoretical dimensions. To assess the reliability and convergent validity of the measurement model, Cronbach’s alpha (CA), Composite Reliability (CR), and Average Variance Extracted (AVE) were calculated. As shown in Table 2, all standardized factor loadings as well as the CA coefficients and CR values exceeded the recommended threshold of 0.70, thus meeting the criteria established in the literature [48]. Convergent validity was further supported by verifying that all AVE values were above the minimum accepted level of 0.50, indicating that the indicators explained a sufficient proportion of the variance of their respective constructs. Additionally, it was confirmed that AVE values were greater than 0.50 and, at the same time, lower than their corresponding CR coefficients [50,51]. Taken together, these results demonstrate that the measurement model exhibits adequate internal consistency and satisfactory convergent validity across all constructs.

Discriminant validity was examined using the HTMT index, considering 0.90 as the maximum acceptable threshold [50,51,52]. As shown in Table 3, all HTMT coefficients are below 0.90 (See Table 3).

Fornell-Larcker criterion [53] was applied, which established that the square root of the AVE values reported on the diagonal of the table must be greater than the correlations with the other constructs in the model. The diagonal values exceeded the interconstruct correlations (See Table 4).

Taken together, these tests (HTMT-Fornell-Larcker criterion) confirmed satisfactory discriminant validity.

### 4.3. Structural Model Assessment (Model Fit and Hypothesis Testing Through Structural Equation Modeling)

After analyzing the psychometric properties of the instrument, the structural model was estimated using SmartPLS 4.1.1.7. The bootstrapping technique with 5000 subsamples was applied to evaluate causal relationships and their statistical significance [54]. To assess the predictive capability of the structural model, the R^2^ values were examined. According to Chin [55], the R^2^ values should exceed 0.10, which is considered acceptable. The following values were obtained: R^2^ for EAW, 0.359; R^2^ for GTR, 0.523; R^2^ for EAT, 0.768; and R^2^ for VCC, 0.738. The R^2^ values indicate that the model explains a substantial proportion of the variance in the endogenous constructs. Specifically, SMC, EAW, and GTR explained 76.8% of the variance in EAT, whereas the model explained 73.8% of the variance in VCC. These results suggest that the proposed framework has strong explanatory power for understanding the mechanisms linking social media communication and value co-creation.

Additionally, the Standardized Root Mean Square Residual (SRMR) was calculated to evaluate the average magnitude of discrepancies between the observed and expected correlations as an absolute measure of model fit. According to Roemer et al. [52], values below 0.10 are typically interpreted as evidence of good model fit; in this study, the SRMR value was 0.08. Furthermore, the results derived from the relationships among the five variables in the proposed model led to the acceptance of eight hypotheses and the rejection of one. The parameter estimates obtained through PLS-SEM indicated that SMC did not have a direct or significant influence on VCC (β = 0.039, *p* > 0.05). However, SMC had a direct and significant influence on the EAT (β = 0.102, *p* < 0.05), EAW (β = 0.599, *p* < 0.001), and GTR (β = 0.723, *p* < 0.001). EAW was found to have a direct and positive influence on VCC (β = 0.382, *p* < 0.001) and EAT (β = 0.470, *p* < 0.001), while GTR exerted a direct and positive influence on VCC (β = 0.369, *p* < 0.001) and EAT (β = 0.382, *p* < 0.001). Finally, this study showed that EAT had a direct and positive influence on VCC (β = 0.391, *p* < 0.001).

As all variables were collected using a single survey instrument at the same time, the potential presence of common method bias was assessed. A comprehensive collinearity assessment was performed using Variance Inflation Factors (VIFs) following the procedure proposed by Kock [56]. The results showed that all VIF values were below the recommended threshold of 3.3, suggesting that common method bias is unlikely to pose a significant threat to the study results. The results are summarized in Table 5.

## 5. Discussion

To facilitate the presentation of the findings, the discussion is structured around the following research questions: (a) How does SMC influence the VCC? (b) How does SMC influence the EAT, EAW, and GTR? (c) How does the EAW influence the VCC and EAT? (d) How does GTR influence VCC and EAT? (e) How does EAT influence the VCC?

### 5.1. Influence of Social Media Content on Value Co-Creation Among Organic Product Consumers

The study found that SMC does not exert a direct influence on VCC, indicating that mere exposure to digital stimuli is insufficient to trigger collaborative behavior among organic product consumers; therefore, H1a is rejected. Although this study does not aim to test a formal mediation model, the results suggest the presence of indirect mechanisms consistent with the SOR framework. Specifically, social media content appears to influence value co-creation primarily through its effects on environmental awareness, green trust, and environmental attitude. These findings indicate that the impact of social media communication is not direct but rather operates through a sequence of cognitive and attitudinal processes that shape consumers’ collaborative behaviors. This interpretation reinforces the theoretical relevance of the SOR model in explaining how digital communication environments translate into value co-creation outcomes. The relevance of this finding lies in challenging the linear assumption that digital interaction automatically leads to shared value outcomes, instead suggesting that co-creation requires the activation of consumers’ internal states, consistent with the SOR model logic that distinguishes stimuli, organisms, and responses [8,29]. From a critical perspective, this result aligns with the literature warning that digital environments do not guarantee participatory behaviors without cognitive and affective processes mediating the transformation of stimuli into behavioral responses [27,28]. However, this contrasts with studies indicating that social media can stimulate shared value creation and sustainable collaboration when digital interactions achieve meaningful consumer engagement [4,18,33]. Thus, the results suggest that in the context of organic consumers, digital content operates more as an indirect trigger than a direct driver of co-creation, reinforcing the idea that co-creation emerges when internal motivations and shared sustainability-related meanings are present [13,15].

### 5.2. Influence of Social Media Content on Environmental Attitude, Environmental Awareness, and Green Truth

The study found that SMC has a direct and significant influence on EAT, indicating that exposure to digital messages related to environmental protection can shape favorable predispositions toward environmental care among organic consumers; therefore, H1b is accepted. This finding confirms that communicational stimuli in digital environments can activate cognitive and affective evaluations associated with environmental care, consistent with the SOR framework [8,29]. This result aligns with the literature suggesting that frequent exposure to environmental content strengthens pro-environmental predispositions when information is perceived as relevant [6,35], while also nuancing arguments that effectiveness depends strictly on source credibility by showing significant attitudinal effects even in organic consumption contexts [5,22]. Hence, social media functions as a space for environmental socialization, capable of consolidating sustainability-oriented dispositions [4,8].

This study also found that SMC significantly influences EAW, showing that exposure to environmental information on digital platforms strengthens the understanding of ecological issues and solutions among organic consumers; therefore, H1c is accepted. This confirms that social media can function as a large-scale environmental literacy channel [36,37]. This finding aligns with studies linking exposure to environmental content with increased pro-environmental intentions [38,39], while also suggesting that informational influence can remain significant, even across heterogeneous contexts [26,39]. Thus, digital environmental content helps build cognitive foundations for interpreting sustainability messages and guiding responsible consumption [8,29].

Additionally, SMC significantly influences GTR, indicating that digital environmental messages help construct perceptions of credibility, authenticity, and coherence. Therefore, H1d is accepted. This confirms that digital communication can strengthen consumer trust when messages are perceived as consistent and aligned with genuine environmental commitments [5,22]. Although consistent with studies emphasizing source and platform trust [23], the results extend the discussion by showing that content itself can reinforce truth judgments when associated with perceived authenticity [8]. Consequently, Green Truth has emerged as a key cognitive mechanism for interpreting sustainability signals in organic markets [10].

### 5.3. Influence of Environmental Awareness on Value Co-Creation and Environmental Attitude

This study found that EAW significantly influences VCC, indicating that greater ecological understanding and sensitivity promote active participation in collaborative sustainability processes; thus, H2a is accepted. This confirms that consumers’ cognitive and value-based foundations are key drivers of shared-value generation. This result aligns with the literature linking environmental literacy to collaborative sustainability dynamics [18,34], while also showing that, in organic consumption contexts, awareness can translate into observable co-creation behaviors [13,15]. Environmentally aware consumers tend to assume active roles when they perceive contributions to collective well-being [14,16].

The study further found that EAW significantly influences EAT, demonstrating that ecological sensitization and understanding translate into favorable predispositions toward environmental protection; therefore, H2b is accepted. This finding supports the view that pro-environmental attitudes are grounded in prior awareness and learning processes. In line with prior studies [20,43], the findings also suggest that digital environments may accelerate awareness consolidation owing to continuous exposure to information [38,39]. Thus, EAW acts as a crucial cognitive antecedent for solid EA [24,25].

### 5.4. Influence of Green Truth on Value Co-Creation and Environmental Attitude

This study found that GTR significantly influences VCC, indicating that when environmental messages are perceived as credible and authentic, consumers become more willing to engage in sustainability-related collaboration; therefore, H3a is accepted. This confirms that communication credibility functions as an intangible resource that strengthens engagement in shared value processes. Although consistent with the literature on trust and collaboration [14,33], it contrasts with arguments about green message saturation leading to skepticism, showing that perceived truthfulness can still translate into co-creation behaviors [22,34]. Thus, GTR acts as a psychological mechanism that legitimizes participation in sustainability initiatives [13,44].

The study also found that GTR significantly influences EAT, demonstrating that perceived authenticity and coherence strengthen favorable environmental predispositions; therefore, H3b is accepted. This shows that EAT depends not only on information but also on trust in message veracity. Aligned with prior research [5,6], the results indicate that GTR can consolidate attitudes even under message saturation [22,35]. Hence, perceived authenticity is a key antecedent to internalizing ecological values [24,25].

### 5.5. Influence of Environmental Attitude on Value Co-Creation

The study found that EAT significantly influences VCC, indicating that favorable environmental predispositions translate into collaborative behaviors within sustainable consumption ecosystems; therefore, H4 is accepted. This aligns with the conceptualization of attitudes as integrating beliefs, emotions, and behavioral intentions that predispose action [24,25]. However, assuming all favorable attitudes lead automatically to co-creation would be simplistic, as literature notes attitude–behavior gaps when contexts do not facilitate action [29,43]. In organic markets, where ecological values and ethical motivations are shared, these gaps appear to be reduced, enabling attitudes to translate into shared-value behaviors [15,18]. The model also shows that EAT is shaped by EAW and GTR, indicating that co-creation results from cumulative value internalization. Thus, the findings reinforce SOR logic by showing that internal states mediate between digital stimuli and collaborative responses in sustainable VCC [27,34].

## 6. Conclusions

This study analyzed the role of SMC in promoting VCC within the context of sustainable organic consumption by integrating the SOR model. The results show that SMC does not directly influence VCC but rather operates through intermediate cognitive and attitudinal processes, particularly EAW, GTR, and EAT. These findings demonstrate that digital communication related to sustainability primarily shapes consumer perceptions and attitudes, which subsequently translate into collaborative behavior. On the other hand, the study contributes to the literature by integrating the areas of social media, sustainable consumption, and VCC within a single explanatory framework based on the SOR model, providing empirical evidence on the role of psychological mechanisms that connect digital communication with active consumer participation. Finally, the results suggest that organizations and retailers using SMC to promote sustainable products should focus their communication strategies on strengthening environmental awareness, building trust in brands’ sustainable practices, and fostering positive attitudes toward responsible consumption, as these factors facilitate consumer participation in the value co-creation processes.

## 7. Theoretical, Practical, and Social Implications

From a social standpoint, the study’s findings generate relevant theoretical, practical, and social implications by demonstrating that sustainable VCC cannot be explained through direct relationships between digital stimuli and behaviors but rather through cognitive and attitudinal mediating processes. This strengthens the contribution of the SOR framework to the field of sustainable consumption and broadens the understanding of how communicative, psychological, and behavioral variables interact in organic markets. From a theoretical perspective, this study contributes to the literature by showing that EAT, EAW, and GTR operate as complementary explanatory mechanisms that help clarify how digital information is transformed into collaborative behavior. From a practical standpoint, the results of this study offer relevant implications for organizations and retailers that use social media to promote sustainable consumption. The findings suggest that SMC does not directly influence VCC but rather acts through cognitive and attitudinal processes such as EAW, GTR, and EAT. Consequently, organizations should design digital communication strategies that not only promote sustainable products but also inform, educate, and build credibility regarding their environmental practices. In this sense, the use of informative content, evidence of sustainable practices, eco-certifications, and spaces for interaction with consumers can strengthen trust and foster active user participation, thus facilitating value co-creation processes in sustainable consumption contexts. From a social perspective, this research suggests that environmental communication on social media can become a collective awareness-raising tool that promotes responsible lifestyles and citizen participation in sustainability, indirectly contributing to environmental and social well-being.

## 8. Limitations and Recommendations for Future Research

The main limitations of the study are as follows: first, the use of a cross-sectional correlational design, which prevents establishing strong temporal causality between SMC, internal states (EAW, EAT, and GTR), and VCC. Consequently, future research should incorporate longitudinal or experimental designs that allow the observation of changes over time and better isolation of the effects of digital stimuli on behavioral responses. Additionally, the sample was obtained through non-probability convenience sampling and consisted of undergraduate and graduate students who reported consuming organic products, which may introduce a selection bias and limit generalizability to other consumer profiles and sociocultural contexts. Therefore, future studies should replicate the model using probability samples, diverse age segments, and different countries or markets to assess external stability.

Furthermore, although the instrument was adapted from prior scales and demonstrated convergent and discriminant validity, some items (EAT4 and VCC4) were removed, which may have affected the full conceptual coverage of the constructs. Thus, future research should refine the scale, revalidate items in new contexts, and further examine potential cultural and semantic equivalence effects. Finally, the model treated SMC in a general manner without distinguishing between content types (informational vs. emotional), content sources (firm-generated vs. user-generated), or platforms (e.g., Facebook, Instagram, and TikTok), which are recognized as potentially influential. Therefore, future research should incorporate moderating variables and multigroup comparisons by platform, content type, and consumer profiles, as well as test the robustness of the model using complementary analytical strategies.

## Figures and Tables

**Table 1:** Demographic characteristics of the respondents.

Characteristics	Category	*n*	%
Gender	Male	310	42%
	Female	429	58%
Age	23 years old or younger	55	7%
	Between 23 and 28 years old	277	37%
	Between 29 and 34 years old	230	31%
	Between 35 and 44 years old	83	11%
	Between 45 y 55 years old	61	8%
	Older than 55 years	33	4%
Education Level	Degree	492	67%
	Postgraduate	247	33%
City	Guayaquil	156	21%
	Quito	188	25%
	Santo Domingo de los Colorados	207	28%
	Cuenca	67	9%
	Other Cities	121	16%

**Table 2:** Convergent validity.

Variable	Item	Factor Loading	CA	CR		AVE
				Rho_a	Rho_c	
Social media content	SMC1	0.768	0.875	0.885	0.914	0.728
	SMC2	0.885				
	SMC3	0.879				
	SMC4	0.877				
Environmental Awareness	EAW1	0.731	0.793	0.847	0.857	0.601
	EAW2	0.756				
	EAW3	0.797				
	EAW4	0.814				
Green Truth	GTR1	0.805	0.889	0.899	0.924	0.753
	GTR2	0.919				
	GTR3	0.915				
	GTR4	0.826				
Environmental Attitude	EAT1	0.932	0.936	0.937	0.959	0.887
	EAT2	0.949				
	EAT3	0.946				
Value Co-creation	VCC1	0.944	0.943	0.944	0.964	0.898
	VCC2	0.959				
	VCC3	0.941				

**Table 3:** Discriminant validity (HTMT).

	SMC	EAW	GTR	EAT	VCC
SMC	-	0.667	0.813	0.726	0.653
EAW		-	0.858	0.860	0.826
GTR			-	0.893	0.877
EAT				-	0.873
VCC					**-**

HTMT ratios positioned above the diagonal (all remaining below 0.90).

**Table 4:** Discriminant validity (Fornell-Larcker criterion).

	SMC	EAW	GTR	EAT	VCC
SMC	0.853				
EAW	0.599	0.775			
GTR	0.723	0.773	0.868		
EAT	0.659	0.726	0.819	0.942	
VCC	0.597	0.771	0.805	0.821	0.948

Fornell and Larcker values displayed on the diagonal and correlation coefficients reported below the diagonal (all remaining below the diagonal values).

**Table 5:** Hypotheses Testing.

Hypotheses	Relation	β	*p*-Value	Hypotheses
H1a	SMC-VCC	0.039	0.268	Rejected
H1b	SMC-EAT	0.102	0.001 **	Accepted
H1c	SMC-EAW	0.599	0.000 ***	Accepted
H1d	SMC-GTR	0.723	0.000 ***	Accepted
H2a	EAW-VCC	0.382	0.000 ***	Accepted
H2b	EAW-EAT	0.470	0.000 ***	Accepted
H3a	GTR-VCC	0.369	0.000 ***	Accepted
H3b	GTR-EAT	0.382	0.000 ***	Accepted
H4	EAT-VCC	0.391	0.000 ***	Accepted

R^2^ EAW: 0.359; R^2^ GTR: 0.523; R^2^ EAT: 0.768; R^2^ VCC: 0.738; SRMR: 0.08; *** *p* < 0.001; *p* < 0.05 ** VIF Values: < 3.3.

**Figure A1:**
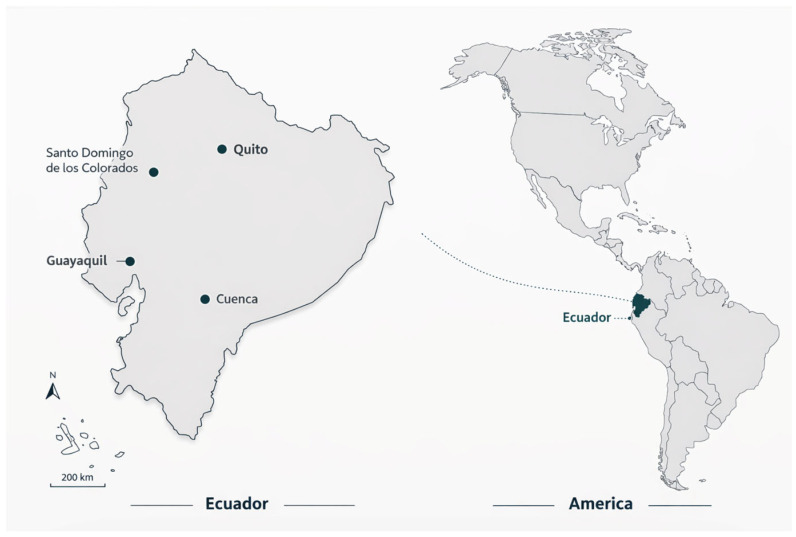
Map of the cities where the study was applied.

## Data Availability

The data used in this study are available upon reasonable request from the corresponding authors.

## Appendix A

### Appendix A.2. Survey Questions

**Table A1:** Items to measure the study variables.

Var.	Item	Author
SMC	**SMC1.** I usually read information and articles about sustainability on social media.	Adapted from: Li et al. [45]
	**SMC2.** I usually see images and videos related to sustainability on social media.	
	**SMC3.** I usually listen to programs on social media related to sustainability.	
	**SMC4.** I usually read government announcements and brochures about sustainable policies and strategies on social media.	
EAW	**EAW1.** I believe that humanity is seriously abusing the environment	Adapted from: Trivedi et al. [46]
	**EAW2.** I think that humans produce disastrous consequences in nature	
	**EAW3.** I consider that the balance of nature is very delicate and easily upset	
	**EAW4.** I think that one must live in harmony with nature in order to survive	
GTR	**GTR1.** I feel that organic food product environmental commitments are generally reliable.	Adapted from: Chen y Chang [47]
	**GTR2.** I feel that organic food product environmental performance is generally dependable	
	**GTR3.** I feel that organic food product environmental argument is generally trustworthy	
	**GTR4.** Organic food product environmental concern meets my expectations	
EAT	**EAT1.** I am very concerned about the environment.	Adapted from: Trivedi et al. [46]
	**EAT2.** I am willing to reduce my consumption to help the environment.	
	**EAT3.** I would contribute financially to help protect the environment.	
	**EAT4.** I have asked my family to recycle some of the things we use.	
VCC	**VCC1.** I use social media to share fresh ideas and information resources.	Adapted from: Li et al. [45]
	**VCC2.** I use social media to take the time needed to discuss new ideas.	
	**VCC3.** I use social media to collaborate and implement new ideas.	
	**VCC4.** When I participate in social media, I feel I have significant autonomy in that participation.	
